# JNK-Bcl-2/Bcl-xL-Bax/Bak Pathway Mediates the Crosstalk between Matrine-Induced Autophagy and Apoptosis via Interplay with Beclin 1

**DOI:** 10.3390/ijms161025744

**Published:** 2015-10-27

**Authors:** Jiong Yang, Shukun Yao

**Affiliations:** 1 Peking University China-Japan Friendship School of Clinical Medicine, Beijing 100029, China; E-Mail: yangjiong@bjmu.edu.cn; 2 Department of Gastroenterology, China-Japan Friendship Hospital, Ministry of Health, Beijing 100029, China

**Keywords:** matrine, JNK, Bax, Bcl-2, Bcl-xL, Beclin 1

## Abstract

Autophagy is associated with drug resistance which has been a threat in chemotherapy of hepatocellular carcinoma (HCC). The interconnected molecular regulators between autophagy and apoptosis serve as switching points critical to the ultimate outcome of the cell. Our study was performed to investigate the crosstalk between autophagy and apoptosis in HCC after the treatment of matrine. Flow cytometry and TUNEL (terminal dexynucleotidyl transferase (TdT)-mediated dUTP nick end labeling) assay were used to detect apoptosis *in vitro* and *in vivo*, respectively. Bax oligomerization and Cytochrome c release assay were performed. Immunoprecipitation and siRNA transfection were used to detect the interplay between Bcl-2/Bcl-xL,Bax, and Beclin 1. Our results showed that: (1) matrine not only activated caspase and PARP (poly ADP-ribose polymerase) cleavage, but also triggered autophagy as shown by the increased levels of LC3II, Beclin 1, and PI3KC3, and the decreased level of p62; (2) matrine treatment promoted the JNK-Bcl-2/Bcl-xL-Bax/Bak pathway; (3) Bax was oligomerized, the mitochondrial membrane potential altered, and Cytochrome c was released subsequently; (4) Bax interacts with Beclin 1 and inhibits autophagy, which may be a new crosstalk point; and (5) finally, we showed that matrine suppressed the growth of a MHCC97L xenograft *in vivo* for the first time. In conclusion, the JNK-Bcl-2/Bcl-xL-Bax/Bak pathway mediates the crosstalk between matrine-induced autophagy and apoptosis via interplay with Beclin 1.

## 1. Introduction

Liver cancer contributed 9.1% to cancer mortality worldwide in 2012, making it the second leading cause of cancer-related deaths [1]. Hepatocellular carcinoma (HCC) accounts for about 90% of all histopathology types of primary liver cancers. During the last decade, although both scientific and clinical advances have been made to improve therapeutic strategies of HCC, prognosis of unresectable cases still remains poor (median survival is less than one year) [2]. Developing new therapeutic strategies is particularly urgent in human HCC.

Apoptosis, the first identified programmed cell death process, has been studied extensively in chemical treatments of tumors [3]. Autophagy, however, is a self-degradative mechanism triggered under stress, especially nutrient deprivation, providing a survival advantage and an association with drug resistance in hepatocellular carcinoma [4]. Accumulating evidence linked them, indicating that their molecular circuitries are not mutually exclusive [5]. The interconnected molecular regulators between autophagy and apoptosis serve as switching points critical to the ultimate outcome of the cell [6,7]. For instance, Bcl-2 and Bcl-xL, the anti-apoptotic proteins. inhibit both autophagy and apoptosis through interacting respectively with Beclin 1 and Bax/Bak using their Bcl-2-homology (BH)-3-binding pockets [8,9,10]. The molecular basis of the interaction between the Beclin 1 and Bcl-2 family proteins is the interaction between the BH3 domain of Beclin 1 and this pocket. Bcl-2/Bcl-xL binds to Beclin 1 and thus prevents the interaction between Beclin 1 and the class III PI3K complex (PI3KC3) to inhibit autophagy [11,12]. Several mechanisms, like JNK-mediated phosphorylation, mediate the disruption of Beclin 1-Bcl-2/Bcl-xL and Bax/Bak- Bcl-2/Bcl-xL interaction [13,14].

In apoptosis, mitochondrial outer membrane permeabilization (MOMP) has been considered as a “point-of-no-return” and is tightly regulated by the well-known Bcl-2 family proteins, especially Bax and Bak [15]. During the process of apoptosis, Bax and Bak translocate to the outer mitochondrial membrane and undergo oligomerization, leading the release of apoptogenetic factors such as Cytochrome c.

Matrine, an alkaloid extracted from *Sophora flavescens*, has been proven to have anti-cancer potential [16]. Our previous work showed that matrine can induce both apoptosis and autophagy in Bel7402 and HepG2 cells [17]. However, the pathways that mediated the crosstalk between matrine-induced autophagy and apoptosis are not clear. In this study, we investigated the effects of matrine-induced autophagy and apoptosis in MHCC97L and Huh-7 cells as well as its underlying crosstalk mechanisms, focusing on the interaction between the JNK-Bcl-2/Bcl-xL-Bax/Bak signaling pathway and Beclin 1. We also used an HCC xenograft tumor model to explore the anti-tumor effects of matrine *in vivo* for the first time.

## 2. Results

### 2.1. Matrine Induces Apoptosis Which Can Be Blocked by Z-VAD-FMK (N-Benzyloxycarbony-Val-Ala-Asp-fluoromethylketone)

**Figure 1:**
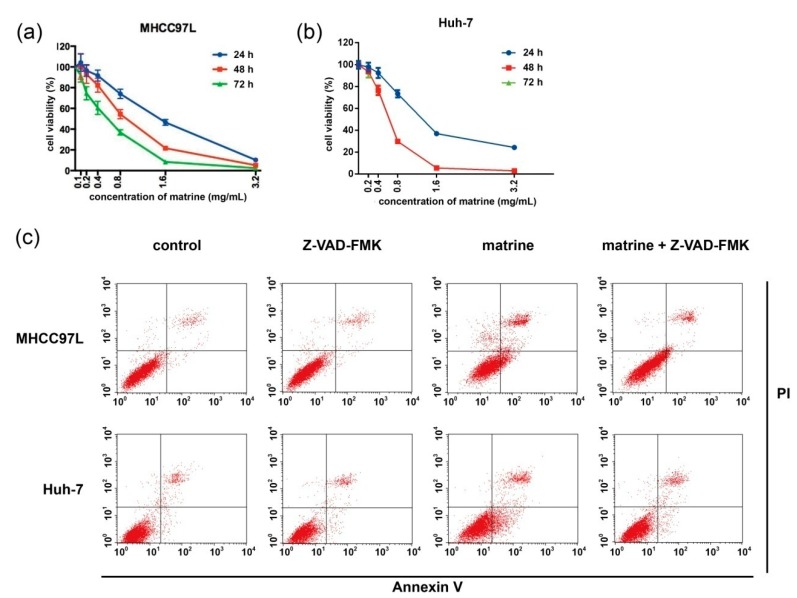

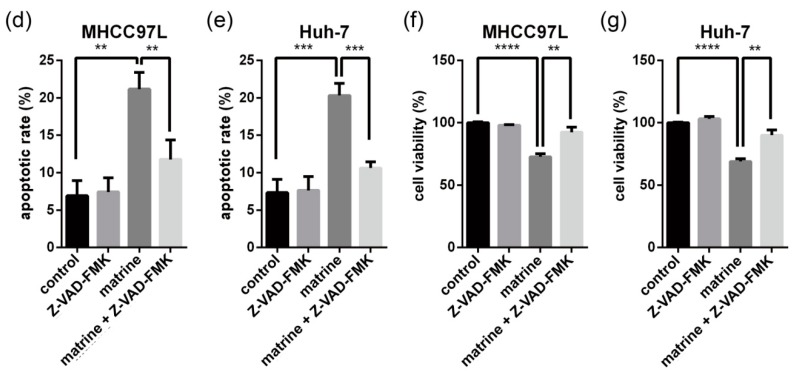
Matrine-induced cytotoxicity in HCC cells. MHCC97L (**a**) and Huh-7 (**b**) cells were treated with a series of concentration gradients of matrine for 24, 48, and 72 h. Then (**a**,**b**) cell viability was analyzed by the CCK-8 cell viability assay; after drug treatments (0.8 mg/mL matrine) for 24 h; MHCC97L and Huh-7 cells (**c**) were stained with PI (propidine iodide) and FITC (fluorescein isothiocyanate) Annexin V. Apoptosis was then detected using flow cytometric assay three times; (**d**,**e**) statistical analysis of apoptotic rate; ** represents a statistically significant difference at *p* < 0.01; *** represents a statistically significant difference at *p* < 0.001, respectively; (**f**,**g**) after drug treatments (0.8 mg/mL matrine, 25 μM Z-VAD-FMK, 0.8 mg/mL matrine + 25 μM Z-VAD-FMK) for 24 h, cell viability was then analyzed by the CCK-8 cell viability assay; **** represents a statistical significance at *p* < 0.0001; *p*-values of ** in (**f**,**g**) were 0.002 and 0.0015, respectively.

To investigate the effects of matrine on cell viability, human hepatocellular carcinoma MHCC97L and Huh-7 cells were treated with a series of concentrations and times of matrine. As the dose and time of matrine increased, cell viability was significantly decreased in both cell lines (Figure 1a,b). Cell viability was restored in the presence of caspase inhibitor Z-VAD-FMK (Figure 1f,g). To clarify whether the cytotoxicity induced by matrine is associated with apoptosis, FITC Annexin V/PI assay was conducted (Figure 1c–e). For flow cytometry assay, MHCC97L and Huh-7 cells were treated with or without matrine and Z-VAD-FMK for 24 h. Apoptotic death cells of late and early stages were observed respectively in the upper right and lower right quadrant of the plots. Our data showed that matrine-induced apoptosis can be blocked by Z-VAD-FMK in both cell lines.

### 2.2. Matrine Induces Autophagy and Caspase Activation

**Figure 2:**
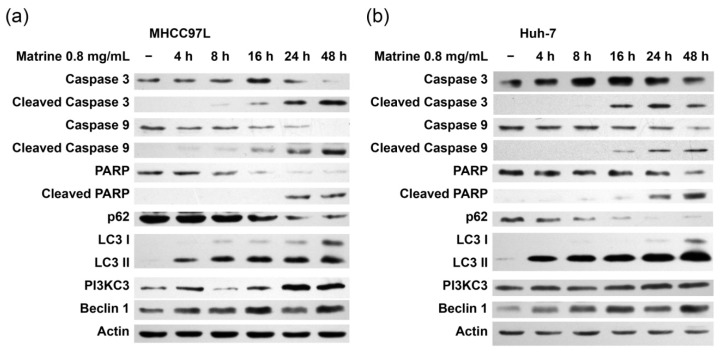
Matrine-induced apoptosis and autophagy in hepatocellular carcinoma cells. MHCC97L (**a**) and Huh-7 (**b**) cells were treated with matrine (0.8 mg/mL) for 4, 8, 16, 24, and 48 h. After matrine treatment, the cleavage of caspase 3, 9, or PARP and p62, LC3, PI3KC3, and Beclin 1 were detected by immunoblotting.

We observed that LC3 lipidation was induced at 4 h after matrine administration, while p62 degradation, the cleavage of caspase 3 and 9, happened at 16 h in MHCC97L and Huh-7 cells (Figure 2a,b). Increased levels of Beclin 1 and PI3KC3 were detected during matrine administration. We also confirmed apoptosis by detecting PARP cleavage, a hallmark of caspase activation. These results suggest that matrine induces apoptosis through activating caspases. Meanwhile, matrine also induced autophagy and upregulated Beclin 1 and PI3KC3 expression.

### 2.3. JNK-Bcl-2/Bcl-xL Pathway Is Activated in Matrine-Induced Autophagy and Apoptosis

**Figure 3:**
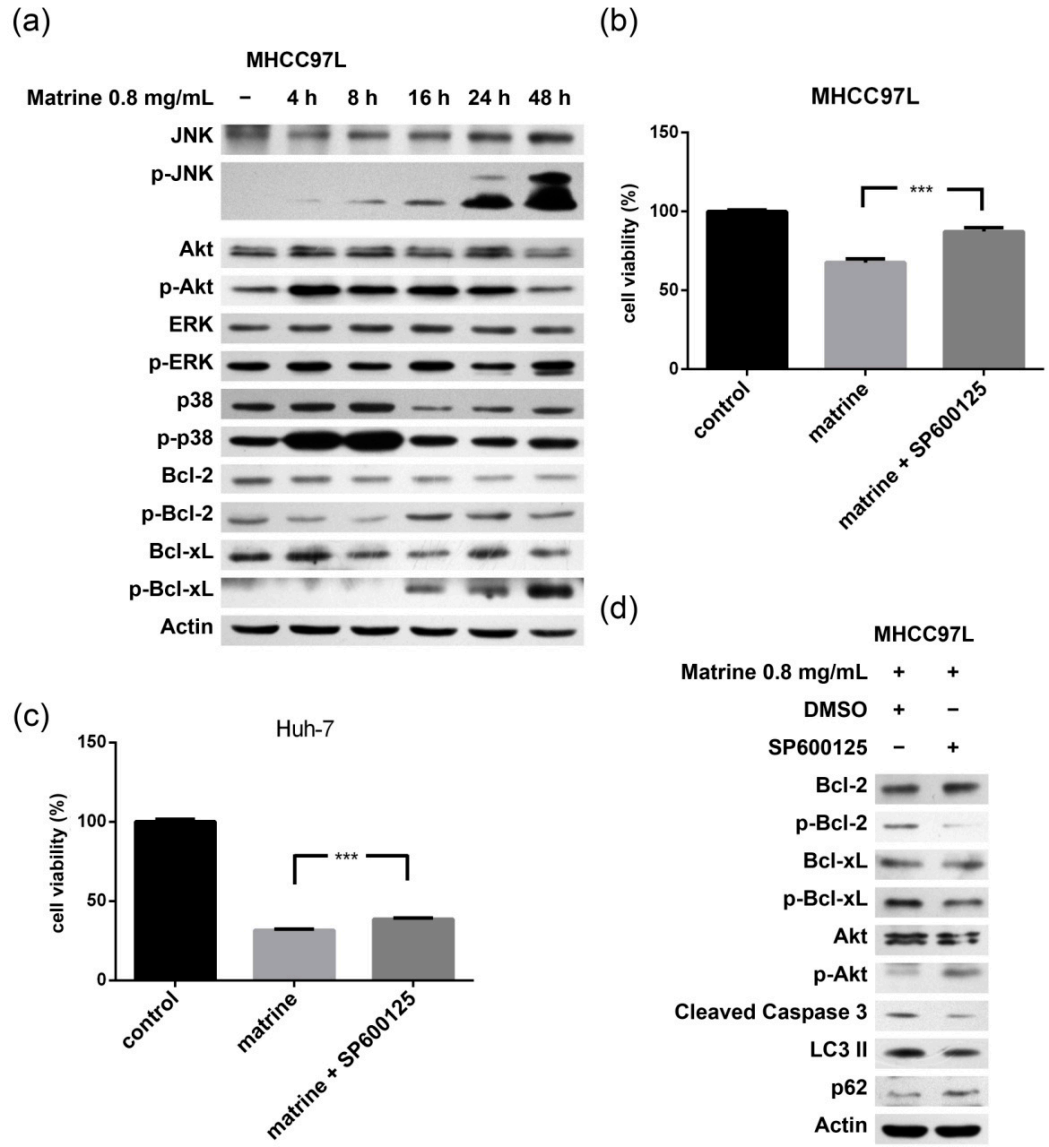
Role of the JNK-Bcl-2/Bcl-xL pathway in apoptosis and autophagy. (**a**) MHCC97L cells were treated with matrine (0.8 mg/mL) for 4, 8, 16, 24, and 48 h. After matrine treatment, proteins in cell lysate were separated by SDS–PAGE. JNK, p-JNK, Akt, p-Akt, p38, p-p38, ERK, p-ERK, Bcl-2, p-Bcl-2, Bcl-xL, and p-Bcl-xL were detected by immunoblotting; (**b**), (**c**) and MHCC97L and Huh-7 cells were treated with matrine (0.8 mg/mL) and matrine (0.8 mg/mL) + SP600125 (90 nM) for 48 h. Cell viability was then analyzed by CCK-8 cell viability assay. *** represents a statistical significance at *p* < 0.001. *p*-Values of (**b**,**c**) were 0.0006 and 0.0003, respectively; and (**d**) After the separation of proteins in cell lysate with SDS–PAGE, Bcl-2, p-Bcl-2, Bcl-xL, p-Bcl-xL, p62, LC3, and the cleavage of caspase 3 were detected by immunoblotting.

The phosphorylation level of JNK, Bcl-2, and Bcl-xL increased during the treatment of matrine while the p-Akt level increased first and then decreased (Figure 3a). The level of p-p38 increased at 4 h but returned to normal at 16 h while p-ERK did not change quite as much (Figure 3a). SP600125, a JNK inhibitor, incompletely restored the viability of MHCC97L and Huh-7 cells that had undergone the matrine treatment (Figure 3b,c). SP600125 treatment reduced matrine-induced LC3 lipidation, p62 degradation, cleavage of caspase 3, and phosphorylation of Bcl-2/Bcl-xL, but increased the level of p-Akt (Figure 3d). These events suggest that JNK is upstream of Bcl-2/Bcl-xL, and the JNK-Bcl-2/Bcl-xL pathway mediated at least part of the matrine-induced autophagy and apoptosis.

### 2.4. Matrine Induces Bax Oligomerization, MOMP, and Cytochrome c Release

**Figure 4:**
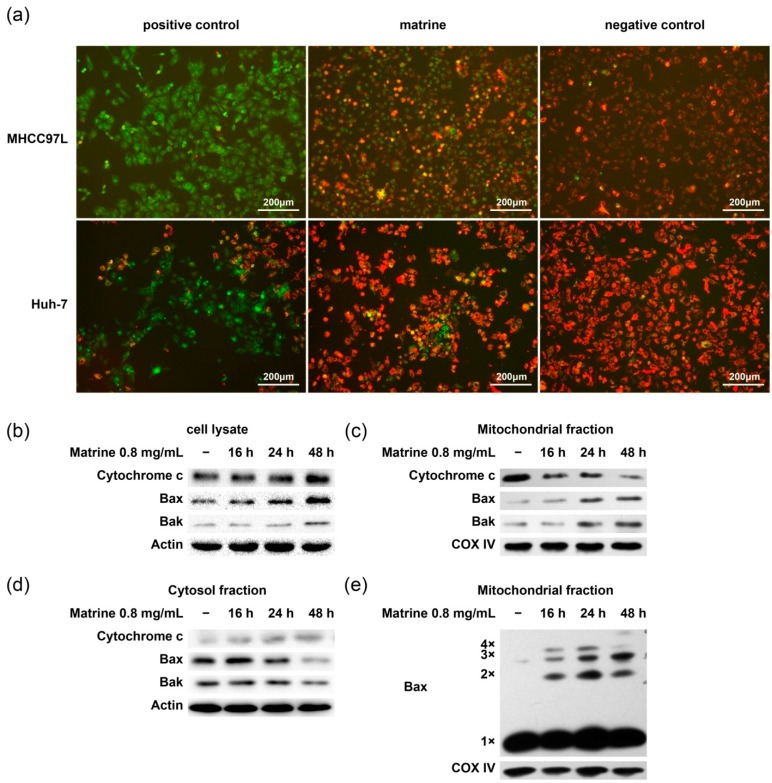
Mitochondrial membrane potential alteration, Bax oligomerization, and cytochrome c release. (**a**) MHCC97L and HuH-7 cells were treated with 10 mM CCCP (carbonylcyanide-*m*-chlorophenylhydrazone) or 0.8 mg/mL matrine for 24 h, stained with JC-1, and then analyzed under fluorescence microscope. Cells treated by CCCP were used as positive control and normal cells were used as negative control; (**b**–**e**) After drug treatment for 16, 24, and 48 h, cell lysate (**b**), cytosolic (**d**) and mitochondrial (**c**,**e**) fractions of MHCC97L cells were obtained. Proteins from each fraction were then preceded to immunoblotting with anti-cytochrome c, anti-Bax, and anti-Bak antibody; (**e**) Bax monomer (1×, 21 kDa) and multimers (2×, 3×, and 4×) were indicated. COX IV was used as the mitochondrial marker and actin as the cytosolic marker.

To further study the effect of matrine on mitochondria, the mitochondrial membrane potential and Bax oligomerization were examined. We observed that the mitochondrial membrane potential decreased after the treatment of matrine in MHCC97L and Huh-7 cells (Figure 4a). Bax oligomerized and translocated from cytosol to mitochondria at 16 h. Bak also translocated to mitochondria. Cytochrome c released from mitochondria to cytosol (Figure 4b–e). These data indicate that matrine upregulated the expression of Bax/Bak, and Bax oligomerized and triggered mitochondria-dependent apoptosis.

### 2.5. Bcl-2/Bcl-xL/Bax Inhibit Autophagy via Interplay with Beclin 1 in Matrine-Induced Autophagy and Apoptosis

**Figure 5:**
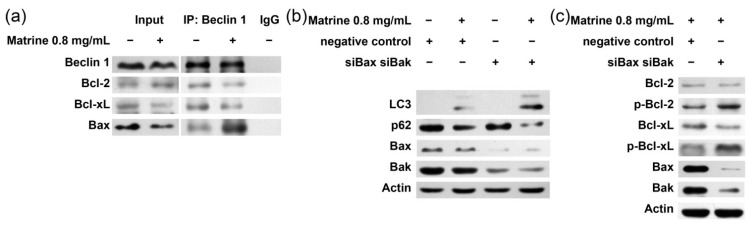
Role of interaction between Bcl-2/Bcl-xL/Bax and Beclin 1 in matrine-induced apoptosis and autophagy. (**a**) MHCC97L cells were treated with matrine (0.8 mg/mL) for 48 h, cell lysates were extracted and immunoprecipitated with anti-Beclin 1 antibody or IgG, and then immunoblotted with anti-Beclin 1, anti-Bcl-2, anti-Bcl-xL or anti-Bax antibodies; (**b**,**c**) MHCC97L cells were transfected with Bax- and Bak-specific siRNAs or negative control for 40 h; (**b**) Cells were treated with or without matrine for 40 h. After being separated by SDS–PAGE, proteins were immunoblotted with anti-p62 and anti-LC3 antibodies; (**c**) Cells were treated with 0.8 mg/mL matrine for 40 h. After being separated by SDS–PAGE, proteins were immunoblotted with anti-Bax, anti-Bak, anti-Bcl-2, anti-p-Bcl-2, anti-Bcl-xL, anti-p-Bcl-xL, anti-p62, anti-LC3 antibodies.

To elucidate the interplay between Bcl-2/Bcl-xL/Bax and Beclin 1 in matrine treatment, a CO-IP assay was performed. Figure 5a shows that after the treatment of matrine, Beclin 1 dissociated from Bcl-2/Bcl-xL and interplayed with Bax. To examine the role of Bax/Beclin 1 interplay in matrine-induced autophagy, MHCC97L cells were pretreated with Bax/Bak siRNA and then stimulated with matrine. The silencing of Bax/Bak not only enhanced autophagy by showing increased levels of LC3 lipidation and p62 degradation, but also increased the phosphorylation of Bcl-2/Bcl-xL (Figure 5b,c). These data suggest that matrine caused dissociation of Bax/Beclin 1 from Bcl-2/Bcl-xL and then Bax bound to Beclin 1 to inhibit autophagy.

### 2.6. Matrine Inhibits the Growth of MHCC97L Xenograft in Vivo

**Figure 6:**
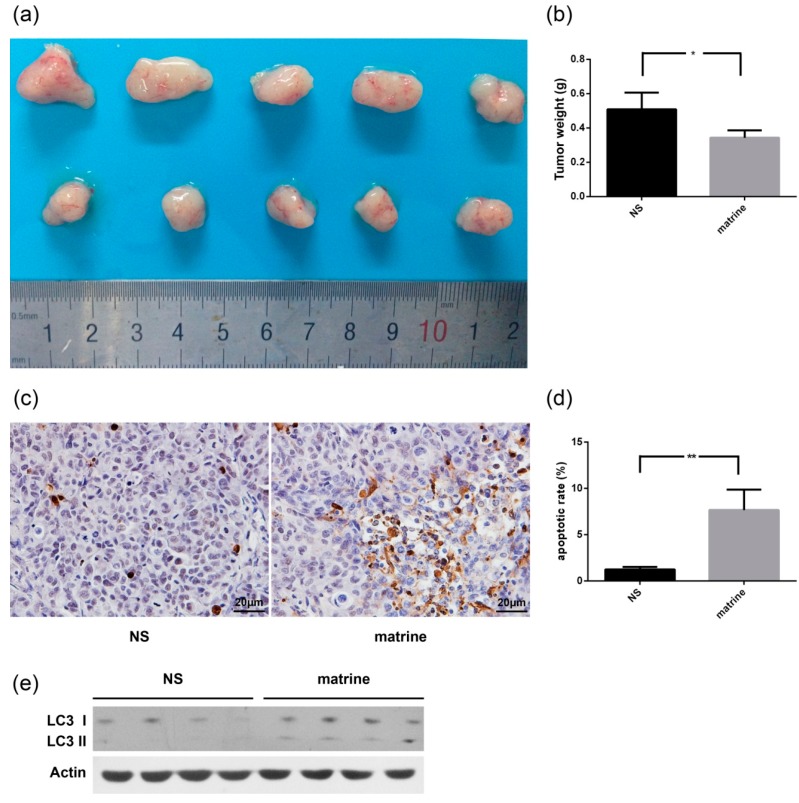
Effect of matrine on the growth of MHCC97L xenograft. Normal saline (NS) or matrine (50 mg/kg) was administered daily by intraperitoneal injection when the tumor volume reached about 200 mm^3^. All mice were killed after four weeks of matrine administration. * represents a statistically significant difference at *p* < 0.05; ** represents a statistically significant difference at *p* < 0.01. (**a**) Photograph of tumor removed from each group; (**b**) Tumor weight of each group (* *p* = 0.0123); (**c**) The apoptotic status of tissues was evaluated by TUNEL assay; (**d**) Apoptotic rate of each group (** *p* = 0.0075); and (**e**) LC3 was detected by immunoblotting.

Preclinical studies were performed to investigate the effect of matrine on the growth of MHCC97L xenografts (Figure 6a). Based on data from four weeks after treatment, matrine caused a statistically significant decrease of tumor growth (Figure 6b). In comparison to the control group, TUNEL assay showed a higher apoptotic rate in tumor tissues of matrine treatment (Figure 6c,d). LC3 was increased after matrine treatment (Figure 6e), indicating that autophagy was also induced *in vivo* (Figure 6e).

## 3. Discussion

Four conclusions can be drawn from our presented data. First, matrine induces autophagy and caspase-dependent apoptosis in HCC cell lines. Second, the JNK-Bcl-2/Bcl-xL pathway is activated and then Beclin 1 and Bax dissociate from Bcl-2/Bcl-xL. Third, released Bax inhibits autophagy by binding to Beclin 1. Finally, matrine treatment significantly inhibits the growth of HCC xenografts.

Autophagy and apoptosis share many molecular regulators. Several pathways have been reported to interfere with autophagy, such as the Akt pathway and MAPK pathways including ERK, p38 MAPK, and JNK [18,19]. JNK has been demonstrated to trigger autophagy by phosphorylating Bcl-2/Bcl-xL and abrogating their bindings to Beclin 1 [20,21]. Akt inhibits JNK activation by negatively regulating several upstream kinases of JNK [22]. Previous studies showed that Bcl-2/Bcl-xL phosphorylation may be a regulatory switch between autophagy and apoptosis [23,24,25]. Figure 3a suggests that matrine has no effect on ERK and has a very short activation on p38 MAPK, but it activates the JNK-Bcl-2/Bcl-xL pathway. After a short time of stimulation, p-Akt is suppressed as well.

Beclin 1, also known as the mammalian homolog of Atg6, is a central regulator of autophagy. It functions as a platform that binds to PI3KC3 to assemble autophagy-inducing complexes [11,26,27]. Our previous work showed that silencing Beclin 1 could enhance matrine-induced apoptosis [17]. Here, we observed that during the treatment of matrine, Beclin 1 is upregulated and dissociates from Bcl-2/Bcl-xL to induce autophagy.

The role Bax plays in autophagy is a debate. Recently, new genetic and biochemical evidence proposed that Bcl-2/Bcl-xL may affect autophagy through its inhibition of Bax and Bak [28,29]. Another study, however, supported the prevailing paradigm by showing that ABT-737 induces autophagy through disruption of the Bcl-2-Beclin 1 complex in cell lines lacking Bax and Bak [30]. Our data showed in Figure 5 suggest that Bax inhibits matrine-induced autophagy by interplaying with Beclin 1. Here we propose a possible explanation. The hydrophobic BH3-binding pocket of Bcl-2/Bcl-xL is formed by BH1, BH2, and BH3 domains. As a member of the Bcl-2 family, Bax also possesses the BH1/BH2/BH3 domains, and the BH3 domain of Bax interacts with the BH1/BH2 domains of another Bax [15]. Thus, Bax possesses the molecular bases for Beclin 1 interaction. After the release from Bcl-2/Bcl-xL, both Bax and Beclin 1 get more opportunity to bind to each other, showing effects of inhibition.

Our data illustrate that matrine activates the JNK-Bcl-2/Bcl-xL-Bax/Bak pathway and effectively triggers apoptosis targeting mitochondria in HCC cell lines. The data also suggest that Bcl-2/Bcl-xL/Bax inhibit autophagy by interplaying with Beclin 1, and mediating the crosstalk between autophagy and apoptosis during the treatment of matrine. The study presented here provides further understanding of matrine, a natural alkaloid, which has chemotherapeutic efficacy on HCC cells both *in vitro* and *in vivo*.

## 4. Experimental Section

### 4.1. Cell Culture

The human hepatocellular carcinoma cell line MHCC97L was obtained from Bioleaf Biotech Co., Ltd., Shanghai, China, and Huh-7 was from the Department of Biochemistry and Molecular Biology, Peking University. Cell lines were cultured in 10% FBS/DMEM (Gibco, Grand Island, NY, USA), 5% CO_2_, at 37 °C.

### 4.2. Reagents and Antibodies

Matrine was purchased from the National Institutes for Food and Drug Control of China and dissolved in DMEM. Anti-β-actin antibody was bought from MBL technologies (Woburn, MA, USA). Anti-Bax, anti-COXIV, anti-PARP, and anti-caspase 3 antibodies were from abcam (Cambridge, MA, USA). Anti-LC3, anti-cytochrome c, anti-cleaved caspase 3, 9, anti-phosphorylated (Thr183) JNK, anti-Akt, anti-phosphorylated (S473) Akt, anti-p38, anti-phosphorylated-p38 (p-p38) antibodies were from Cell Signaling (Danvers, MA, USA). Anti-p62/SQSTM1 antibody was from Biosynthesis Biotechnology (Beijing, China). Anti-JNK, anti-ERK, anti-phosphorylated-ERK (p-ERK), anti-Bcl-2, anti-phosphorylated (Ser87) Bcl-2, anti-Bcl-xL, anti-phosphorylated (Ser62) Bcl-xL, anti-Bak, anti-caspase 9 antibodies were from Affinity (Cincinnati, OH, USA). Anti-PI3KC3 antibody was from Abgent (San Diego, CA, USA). Anti-Beclin 1 antibody, SP600125 (dissolved in DMSO), and Z-VAD-FMK were from Santa Cruz (Santa Cruz, CA, USA).

### 4.3. Survival Assay

Cells were seeded in 96-well cell culture plates at a density of about 2 × 10^3^ cells per well. After drug treatments, CCK-8 assay (Dojindo Molecular Technologies Inc., Kumamoto, Japan) was performed following the instructions from the manufacturer.

### 4.4. Flow Cytometry Analysis

Cells were seeded in six-well cell culture plates, and then treated with drugs for 24 h. Following the manufacturer’s instructions, cells were stained with FITC Annexin V Apoptosis Detection Kit I (BD Biosciences, San Jose, CA, USA). Apoptosis was analyzed by flow cytometry (FACS Aria, BD Biosciences).

### 4.5. Western Blot Analysis

Total cellular protein was extracted with RIPA (25 mM Tris–HCl, pH 7.5, 150 mM NaCl, 1% Nonidet P-40, 1% sodium deoxycholate, 0.1% SDS) lysis buffer containing protease inhibitor PMSF. Protein amount in the lysate was measured using Pierce BCA Protein Assay Kit (Thermo Scientific, Rockford, IL, USA). After being boiled for 5 min, protein samples were subjected to SDS–PAGE, electrophoretically transferred to nitrocellulose membranes under proper conditions, and blocked with 5% BSA or 5% skim milk dissolved in TBS-Tween 20 (0.1%, *v*/*v*) for 2 h. Primary antibodies were incubated with corresponding membranes at 4 °C overnight, and then incubated with secondary antibodies at room temperature for 2 h. The protein bands were visualized by a Western Lightning Plus ECL (Thermo Scientific).

### 4.6. JC-1 (5,5,6,6-Tetrachloro-1,1,3,3-tetraethylbenzimidazolylcarbocyanine iodide) Mitochondrial Membrane Potential Assay

Cells were stained with JC-1 in Mitochondrial Membrane Potential Assay Kit (Beyotime, Beijing, China) after drug treatment. Stained cells were washed twice before observing under the fluorescence microscope (Thermo Scientific, West Palm Beach, FL, USA).

### 4.7. Bax Oligomerization

After drug treatment, cells in each group were collected and resuspended in homogenization buffer for 10–15 min. The cell suspension was centrifuged at 4 °C, 1000× *g*, 10 min to obtain nuclear pellets which would be discarded in the next step. Supernatant containing mitochondria was transferred to a new eppendorf tube and centrifuged at 4 °C, 10,000× *g*, 15 min to pellet the mitochondria. Isolated mitochondrial and cytosolic fractions were cross-linked with 1 mmol/L dithiobis (Pierce) for 1 h at room temperature. Mitochondrial proteins were subjected to immunoblotting for Bax. COX IV was chosen to be the mitochondrial marker.

### 4.8. Cytochrome c Release Assay

After drug treatment, about 5 × 10^7^ cells were collected. Mitochondrial and cytosol fractions were obtained by using Cell Mitochondria Isolation Kit (Beyotime), following the steps in the instructions. Cytosolic fractions were subjected to immunoblotting assay using anti-cytochrome c antibody.

### 4.9. Small Interfering RNA Transfection

Human Bax- and Bak-specific siRNAs (GenePharma, Suzhou, China) were transfected into MHCC97L cells using Lipofectamin 2000 reagent. Control group was transfected with a nontargeting siRNA. The knocking down efficiency of respective proteins was checked by Western blotting. Sequences of siRNAs: Bak (5ʹ-GGAGCUGCAGAGGAUGAUUTT-3ʹ), Bax (5ʹ-GGUCACCUUACCUCUGCAATT-3ʹ), negative control (5ʹ-UUCUUCGAACGUGUCACGUTT-3ʹ).

### 4.10. Immunoprecipitation

Cells were collected and lysed with lysis buffer (50 mM Tris-HCl, pH 7.5, 150 mM NaCl, 2 mM EDTA, 0.5% NP-40) and PMSF. Then 500 μg lysate was incubated with 1 μg rabbit anti-Beclin 1 antibody or IgG (rabbit) and agarose beads (protein G), rotating at 4 °C overnight. Immunoprecipitates were collected by centrifugation at 500× *g*, 5 min, and the pellet was mixed with 2× loading buffer for heat denaturation. After centrifugation, the supernatant was subjected to SDS–PAGE and immunoblot analysis.

### 4.11. Animal Model

The subcutaneous xenograft model was established by injecting 1 × 10^6^ MHCC97L cells subcutaneously into the right axilla of each five-week-old male Balb/c nude mouse (Department of Laboratory Animal Science, Pecking University Health Science Center, Number SCXK 2011-0012 of qualitive qualification, Number of permit: SYXK 2011-0039, Term of validity: from 12 September 2011 to 12 September 2016). After establishment of these xenografts, mice were randomized into two groups of five mice per group. Matrine injection (Guangzhou Baiyun Shan Ming Xing Pharmaceutical Co., Ltd., Guangzhou, China) and normal saline were administered by intraperitoneal injection. Mice were maintained in SPF (specificpathogen free) environments and fed by breeders in the Department of Laboratory Animal Science, Pecking University. All the animal studies performed here were both reviewed and approved by the Ethics Committee for Animal Studies at Pecking University, China.

### 4.12. TUNEL (Terminal Dexynucleotidyl Transferase (TdT)-Mediated dUTP Nick end Labeling) Assay

A TUNEL *in situ* cell apoptosis detection kit (KeyGEN BioTECH, Nanjing, China) was used for the detection of apoptotic cells *in situ*. The staining was performed following manufacturer’s instructions. Slides were deparaffinized, hydrated, washed, permeabilized, and incubated with TUNEL reaction mixture. After washing, slides were observed under optical microscope (Bio-Rad, Foster, CA, USA).

### 4.13. Statistical Analysis

The quantitative data were presented as means ± SD, and were analyzed by prism 6 (GraphPad, San Diego, CA, USA). *p*-Values were determined by Student’s *t*-test and *p* < 0.05 was considered having statistical significance.
